# The effectiveness of an integrated continuous team midwifery care model on the quality of antenatal care in Iran’s health system: A randomized controlled trial

**DOI:** 10.1016/j.eurox.2025.100420

**Published:** 2025-08-07

**Authors:** Farzaneh Nasiri, Azam bagheri, Maryam Dastoorpour, Shahla Khosravi, Zahra Abbaspoor

**Affiliations:** a Department of Midwifery, Reproductive Health Promotion Research Center, Ahvaz Jundishapur University of Medical Sciences, Ahvaz, Iran; b Department of Midwifery and Reproductive Health, Kashan University of Medical Sciences, Kashan, Iran; c Department of Biostatistics and Epidemiology, School of Public Health, Ahvaz Jundishapur University of Medical Sciences, Ahvaz, Iran; d Department of Community Medicine, School of Medicine, Tehran University of Medical Sciences, Tehran, Iran

**Keywords:** Midwifery, Continuity, Antenatal, Quality, Pregnancy, Randomized controlled trial

## Abstract

**Background and aims:**

In maternity care, high quality prenatal care is essential for both women and babies. Continuity and coordination of midwifery care are very crucial in improving the quality of care. The present study investigated the effectiveness of an integrated continuous team midwifery care model (ICTMC) on the quality of antenatal care in Iran’s health system.

**Methods:**

This randomized controlled trial included 200 low-risk nulliparous women selected from governmental health centers in Kashan, Iran during 2023–2024. The women were randomized into two groups, with block sizes of four and six, maintaining a 1:1 allocation ratio. A dedicated team of midwives offered continuous care throughout the gestation, labor and parturition periods plus postnatal care (6 weeks after birth) for the women in the intervention group. The control group, on the other hand, received standard care from various midwives during the same periods. A demographic questionnaire, and the Quality of Prenatal Care Questionnaire were used for data collection. Data were analyzed using descriptive and inferential statistics in STATA-17

**Results:**

The ICTMC group exhibited a significantly higher mean of quality of prenatal care scores compared to the control group (158.83 ± 17.13 vs.133.51 ± 24.48, p < 0.001), and this superiority was observed in all dimensions of this scale.

**Conclusion:**

The present study offers evidence for the benefits of ICTMC, A collaborative approach among inform policymakers, healthcare providers, and researchers is recommended to facilitate the implementation of ICTMC within the Iranian healthcare system.

## Highlights

• Investigated continuous team midwifery care model in Iran's fragmented maternity care system.
• Continuous team midwifery care model can implemented in integrated health systems.
• Continuous team midwifery care model improves quality of maternal care significantly.
• Integrated continuous team midwifery care model Promising approach for enhancing maternal care in Iran.

## 1 Introduction

Optimal maternal and neonatal outcomes are contingent upon receiving excellent maternity care throughout gestation, parturition, and the postpartum periods [1]. To be considered truly high-quality, maternity care must encompass a multitude of elements. These include efficiency in service delivery, effectiveness in achieving positive health outcomes. Additionally, care should be readily available, comprehensive in its scope, and respectful of individual preferences. Timeliness, appropriateness of interventions, and continuity of care by a familiar provider are also crucial. Finally, patient privacy and confidentiality must be upheld throughout the entire process [2]. In health services lack of an ongoing relationship over time cannot be compensated for by continuity of management and information. Indeed, continuity and coordination of care through integrated, person-centered health services are very important for enhancing the quality of care [3].

As far as maternity care is concerned, good quality antenatal care is essential for both women and babies [1]. Research suggests a significant shortfall in access to quality maternal care, with as few as 20 % of pregnant women receiving adequate prenatal attention [4]. Compounding the issue of limited access, a prior study revealed a concerning trend of women initiating prenatal care in the second or third trimester. This delay coincides with a critical window for monitoring fetal development and addressing potential complications [5]. It has also been reported that 83 % of pregnant women have received average and insufficient care during pregnancy [6].

Low quality of prenatal care is associated with increased infant mortality and adverse neonatal outcomes [7], [8]. Also, as the quality of care decreases, unnecessary interventions increase for mothers, and the health of mothers is compromised [9], [10].

One promising approach to enhancing the quality of care for both mothers and newborns is the implementation of midwifery-led care models [11], [12], Midwifery-led models prioritize individualized care, tailoring services to each woman's specific needs. When necessary, these models facilitate seamless collaboration with other specialists to ensure comprehensive care, and midwives are in active partnership with women who actively participate in their own needs, plan care and refer to others if needed [13].

One type of midwifery-led care models is continuous team midwifery care. This model features a cohesive team of midwives working collaboratively within a midwifery group practice (MGP). Women benefit from receiving care throughout gestation, parturition, and postnatal periods from this consistent team of midwives [14]. An updated systematic review has found compelling evidence that these models reduce the use of medical interventions, improve neonatal health outcomes, and enhance maternal satisfaction with care [12].

In a clinical trial conducted in Australia, Allen et al. showed that continuous midwifery care has more quality prenatal care than do routine care and quality standards [15]. On the other hand, a number of studies have reported conflicting results. Aune et al., for instance, found that continuity of care did not make a difference to birth outcomes [16].

Although prenatal care is provided in governmental health centers in Iran, there is not continuity in the healthcare system in providing care [17]. A critical concern within the Iranian healthcare system involves the fragmentation of care, particularly the lack of communication between governmental perinatal midwifery clinics and the labor and postnatal teams [18].

Previous studies, such as the study by Shahinfar et al. demonstrated the positive effects of team midwifery care on maternal and neonatal outcomes; however, as this study was conducted in the private sector, its findings may not be generalizable to the public healthcare system in Iran [19].

Similarly, the study by Bagheri et al., evaluated Comparing the Implications of Midwifery-Led Care and Standard Model on Maternal and Neonatal Outcomes during Pregnancy, Childbirth and Postpartum but it did not focus on measuring antenatal care quality [20].

This has reduced the quality of care. There appears to be a lack of research investigating the implementation of ICTMC within Iranian governmental health centers to improve the quality of pregnancy care. To address this problem, the present study aimed to evaluate the effectiveness of an integrated continuous team midwifery care model on the quality of antenatal care in Iran’s health system.

## 2 Materials and methods

This randomized controlled trial was conducted as part of a comprehensive and extensive PhD dissertation project. Due to the broad scope and multiple outcome measures of the study, the present manuscript focuses exclusively on reporting the results related to the quality of antenatal care.

The study recruited 200 pregnant women in Kashan, Iran, and divided them into two comparable groups. The study adhered to CONSORT guidelines. Data collection occurred between April 30th, 2023, and January 20th, 2024. All participants provided written informed consent before participation. All procedures were conducted in accordance with the ethical standards of the institutional research committee and the Declaration of Helsinki. Confidentiality and anonymity of participants’ data were strictly maintained throughout the study.

The study included only married Iranian women aged 18–35 expecting their first child (primiparous), with a low-risk singleton pregnancy at ≤ 12 weeks gestation and possessing basic literacy. Exclusion criteria included a history of medical disorders during pregnancy (such as diabetes, hypertension, cardiovascular disease, renal disease, hepatic disease, or brain disease), undergoing cervical cerclage during the study, or a history of abortion.

### 2.1 Setting

The research was conducted across 15 public health within Kashan University of Medical Sciences. These designated centers were specifically chosen because they provided care for women throughout pregnancy and after delivery, making them ideal for the study's sampling process. The study specified a target enrollment of eight to sixteen women per center. Deliveries were scheduled to take place at two hospitals affiliated with Kashan University of Medical Sciences: Shahid Beheshti and Shabihkhani Hospitals.

### 2.2 Sample size

Following Shahinfar et al. [19], the target sample size was calculated to be 100 participants per group. The calculation was based on mean values of 291.44 and 212.62, standard deviations of 44.76 and 62.04, a significance level (α) of 0.05, and a statistical power of 90 %.

### 2.3 Randomization

The method of assigning women to the intervention or control groups was based on block randomization with a block size of 4 and 6, and an allocation ratio of 1:1. Allocation concealment was ensured using a system of sequentially numbered, opaque, sealed envelopes prepared by an independent third party who was not involved in participant recruitment, data collection, or aware of the study objectives. These envelopes, containing the group assignments, were opened only after informed consent was obtained, thereby preventing foreknowledge of allocation during enrollment.

Due to the nature of the intervention, blinding of participants and care providers was not feasible. Additionally, as the Quality of Prenatal Care Questionnaire was assessed through participants' self-reported responses using the questionnaire, participant blinding was not possible. However, outcome data analysts remained blinded to group allocation throughout the study

### 2.4 Intervention and follow up

Women assigned to the ICTMC intervention received comprehensive antenatal, intrapartum, and postpartum (including six weeks postnatal) care from a dedicated midwifery team. This team included five members: the lead researcher (FN), two midwives from the participating health center, and two midwives from the collaborating hospital.

In each health center, two midwives were assigned to each pregnant woman in the intervention group (one as the main midwife and one as a backup midwife). If the primary midwife was unavailable, the woman would contact the back-up midwife.

In each hospital, there were two midwives (as the main and backup midwives) on each shift work. It should be noted that the pregnant women had prior familiarity with the hospital midwives through childbirth preparation classes and hospital visits. The lead researcher (FN) maintained a high level of presence throughout the prenatal, intrapartum, and postnatal care periods. Midwives from participating health centers delivered this antenatal care and postnatal care from 24 h after delivery up to 6 weeks postpartum. Hospital midwives, also members of the team, managed labor, delivery, and immediate postpartum care (up to 24 h after delivery).

All women received three standardized obstetric consultations scheduled at key gestational ages: 12–16, 28, and 36 weeks. For pregnancies extending beyond 41 weeks gestation, an additional obstetric consultation was provided. The midwife team maintained a collaborative approach, working with obstetricians and other healthcare providers to address any complications that might arise.

Eight 2-h sessions of childbirth preparation classes were conducted by the midwifery team. Beyond adhering to Iran's national pregnancy care protocol, the intervention group received an additional service. During the initial prenatal visit, the women were provided with a 24/7 phone number for unrestricted communication regarding questions or concerns. An online group was established, facilitating access to educational materials related to pregnancy, childbirth, and women's choices during this process.

Women in the control group were given routine care by the existing healthcare providers within the facility. This included care from different midwives and obstetricians as per the availability of these professionals. Also, there was no continuity of care in this group. Pregnant women encountering questions or problems beyond these designated times lack a designated point of contact within the system. Their options are limited to seeking care at private hospitals or clinics, where no established connection exists between the midwives and the women, they previously see at health centers. Notably, health center midwives are not informed when their patients are admitted. In our study, the control group received care adhering to the same national guidelines and protocols as the intervention group.

### 2.5 Outcome

Outcome was the quality of prenatal care, assessed using the validated Quality of Prenatal Care Questionnaire (QPCQ), which was self-completed by all participants after 36 weeks of gestation.

### 2.6 Instruments

#### 2.6.1 Demographic questionnaire

The demographic questionnaire was administered through face-to-face interviews conducted at the study's outset to collect baseline information before randomization

#### 2.6.2 Quality of prenatal care questionnaire (QPCQ)

QPCQ was developed by Heaman et al. in Canada [21] to assess participants' perceptions of their prenatal care experiences. The QPCQ utilizes a 5-point Likert scale. The total score range is 46–230. There are 6 subscales in this instrument: Availability; Sufficient Time; Information sharing; Approachability; Anticipatory Guidance; and Approach and Respect. Higher scores, both for the total questionnaire and individual subscales, reflect participants' perception of higher quality prenatal care. The instrument's validity and reliability have been established and approved for use in the Iranian population [22], [23]. Reliability was further supported by the Cronbach's alpha coefficient (0.883) and ICC (0.822) of the entire questionnaire [23].

### 2.7 Statistical analysis

This study used descriptive statistics (frequency, mean, standard deviation) and Skewness and kurtosis were assessed for the variables. The values fell within the acceptable ranges (skewness: −2 to +2; kurtosis: −7 to +7), indicating that the data were approximately normally distributed. The study used different statistical tests depending on the type of data and group comparisons. Chi-square test for trend were used for categorical data, while independent samples *t*-test was used for numerical data. Stata version 17 was used, with a significance level set at less than 0.05.

## 3 Results

**Fig. 1:**
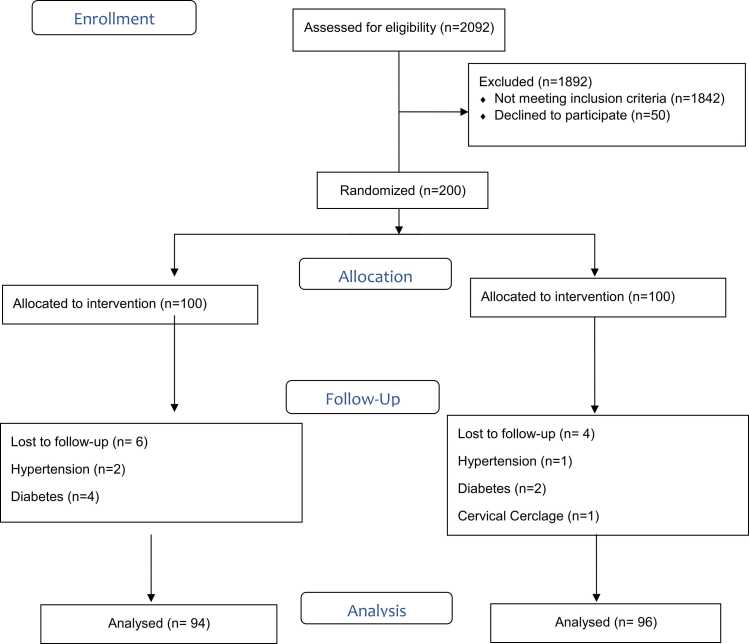
Flow-diagram.

Out of 2092 women screened between April 2023 and January 2024, 200 (9.5 %) met all eligibility criteria and consented to participate. The remaining 1892 women (90.5 %) were excluded, with 1842 (88 %) not meeting inclusion criteria and 50 (2.4 %) declining participation. Following randomization, 100 participants were allocated to the ICTMC group and 100 to the control group (routine care). Six participants (6 %) from the control group and four participants (4 %) from the intervention group dropped out of the study. The intention-to-treat analysis included data from 96 women in the intervention group and 94 women in the control group. (Baseline demographic and obstetric variables of the study participants Fig. 1).

Table A 1 presents the baseline characteristics of the women studied. The average age for women in both the intervention and control groups was around 26 years old (26.61 ± 4.22 years in intervention vs. 26.19 ± 3.92 years in control), with no significant statistical difference (p = 0.467). Education levels women showed no significant differences between the groups (p = 0.600¥).

Women with ICTMC had higher mean of quality of prenatal care compared to women without an ICTMC (202.15 ± 18.39 vs. 153.67 ± 28.81, p < 0.001), suggesting that ICTMC led to demonstrably better prenatal care across all dimensions measured (Table A 2).

No serious adverse events were reported in either group. No participants discontinued the intervention due to harms.

## 4 Discussion

The present study investigated The effectiveness of an integrated continuous team midwifery care model (ICTMC) on the quality of antenatal care in Iran’s health system.

The findings showed that ICTMC is associated with increased quality of prenatal care and significantly enhances all its dimensions of quality of prenatal care.

The quality of healthcare delivery is a paramount concern in healthcare systems, as it demonstrably impacts patient outcomes [24]. Furthermore, the quality of care during pregnancy holds particular significance. A growing body of research demonstrates a clear association between diminished quality of prenatal care and poorer maternal and neonatal outcomes [8]. Our study's findings align with a Cochrane review, indicating that the ICTMC model achieved higher ratings across all quality dimensions of prenatal care. This is consistent with the review's conclusion that women receiving midwife-led care models, like ICTMC, experience a higher standard of care [25]. Abedi et al. posited that continuous midwifery care (ICTMC) represents a key strategy for enhancing the quality of midwifery services in Iran [26]. Because in continuous midwifery care, the relationship between midwives and pregnant women is stronger, the support of pregnant women is greater, and they will be empowered and engaged in decision-making [27].

The present study was conducted in health centers and public hospitals, where there was no continuity in services. The results of this study suggest that a model like ICTMC, which provides continuous midwifery care throughout pregnancy, childbirth, and postpartum periods, could be a promising strategy to address the needs of Iranian women. This approach might be linked to a higher standard of prenatal care in this population.

Similar to other regions [28], Iran's healthcare system faces challenges in implementing midwifery continuity of care models. Organizational culture, power dynamics between professions, financing structures, and integration issues within the healthcare system present as significant barriers. To address this gap and improve women's health outcomes throughout pregnancy, childbirth, and postpartum care, a shift in policy focus is necessary. Specifically, government support for ICTMC implementation, particularly during labor and delivery, could be a viable solution to combat Iran's rising cesarean section rates.

### 4.1 Strengths and limitations

A key limitation of this study is the recruitment setting. Conducted solely within public hospitals and centers, the generalizability of the findings to private hospitals might be limited. However, our study has several strengths. Firstly, it employed a randomized sampling method, enhancing the internal validity of the results. Secondly, to the best of our knowledge, it represents the first investigation into the impact of team midwifery care on prenatal care quality for low-risk nulliparous women in Kashan, Iran, contributing valuable new knowledge to the field.

## 5 Conclusion

This study investigated ICTMC in the fragmented maternity care system in Iran. Our results showed that this model can be easily implemented in the health system of countries like Iran, where basic health services are integrated and widely provided. ICTMC has the potential to significantly improve the quality of maternal care in these settings. Implementing ICTMC within the Iranian healthcare system holds significant promise for enhancing maternal care quality.

## CRediT authorship contribution statement

**nasiri farzaneh:** Writing – original draft, Visualization, Validation, Resources, Project administration, Methodology, Investigation, Funding acquisition, Formal analysis, Data curation, Conceptualization. **Bagheri Azam:** Writing – review & editing, Visualization, Supervision, Project administration, Methodology, Investigation, Formal analysis, Conceptualization. **Maryam Dastoorpour:** Writing – review & editing, Validation, Software, Methodology, Formal analysis, Conceptualization. **Shahla Khosravi:** Writing – review & editing, Visualization, Supervision, Project administration, Methodology, Investigation, Conceptualization. **Zahra Abbaspoor:** Writing – review & editing, Visualization, Validation, Supervision, Resources, Project administration, Methodology, Investigation, Formal analysis, Conceptualization.

## Ethical considerations

This paper reports the findings of the research study that adhered to the Declaration of Helsinki.

This study received ethical approval from the Ethics Committee of Ahvaz Jundishapur University of Medical Sciences, under the ID IR.AJUMS.REC.1402.010. Additionally, it was registered with the clinical trial code IRCT20171106037279N4.

## Funding

This study was supported by 10.13039/501100005001Ahvaz Jundishapur University of Medical Sciences, Ahvaz (Grant ID: 330101336).

## Declaration of Competing Interest

The authors declare that they have no known competing financial interests or personal relationships that could have appeared to influence the work reported in this article.

## Figures and Tables

**TableA. 1:** Baseline demographic and obstetric variables of the study participants

**Variables**	**Continuous Team Midwifery Care group** **(n = 100)**	**Control group** **(n = 100)**	**p-value**
	**Mean ± SD/** **n(%)**	**Mean ± SD/** **n(%)**	
Woman’s age (years)	26.61 ± 4.22	26.19 ± 3.92	0.467*
Husband’s age(years)	30.8 ± 4.07	30.27 ± 3.87	0.347*
Body mass index)kg/m2)	23.94 ± 2.22	23.98 ± 1.96	0.879*
**Woman’s education level**			
Primary or secondary School	4 (4)	3 (3)	0.600¥
High school diploma	49 (49)	43 (43)	
University degree	47 (47)	54 (54)	
**Husband’s education level**			
Primary or secondary School	5 (5)	6 (6)	
High school diploma	55 (55)	51(51)	0.841¥
University degree	40 (40)	43 (43)	
**Employment status**			
Housewife	79 (79)	82 (82)	0.592**
Employed	21 (21)	18 (18)	
**Economic status**			
Poor	10 (10)	11 (11)	0.937**
Moderate	82 (82)	80 (80)	
Good	8 (8)	9 (9)	
**Nationality**			
Fars	23 (23)	27 (27)	0.514**
Non-fars	77 (77)	73 (73)	

* Independent samples *t*-test

** Chi-square test

¥ Chi-square test for trend

**Table A.2:** Quality of prenatal care in the study groups

**Characteristics**						
		Mean ± SD	Score Range*	Mean Differences	95 % confidence interval	p-value**
Information sharing	**Intervention group(n = 96)**	40.87 ± 3.55	9–45	−8.40	(−9.73- −7.06)	< 0.001
	**Control group (n = 94)**	32.47 ± 5.58				
Anticipatory Guidance	**Intervention group(n = 96)**	47.73 ± 4.65	11–55	−12.20	(−13.92- −10.48)	< 0.001
	**Control group (n = 94)**	35.54 ± 7.11				
Sufficient Time	**Intervention group(n = 96)**	21.85 ± 2.32	5–25	−3.11	(−3.81 - −2.41)	< 0.001
	**Control group (n = 94)**	18.74 ± 2.53				
Approachability	**Intervention group(n = 96)**	15.34 ± 4.08	20–4	−5.12	(−6.42 - −3.82)	< 0.001
	**Control group (n = 94)**	10.22 ± 4.99				
Availability	**Intervention group(n = 96)**	22.93 ± 2.67	5–25	−9.20	(−10.39- −8.01)	< 0.001
	**Control group (n = 94)**	13.73 ± 5.26				
Support and respect	**Intervention group(n = 96)**	53.40 ± 5.23	60–12	−10.46	(−12.15 - −8.76)	< 0.001
	**Control group (n = 94)**	42.94 ± 6.54				
Total Quality Of Prenatal Care	**Intervention group(n = 96)**	202.15 ± 18.39	46–230	−48.49	(−55.38951- −41.58257)	< 0.001
	**Control group (n = 94)**	153.67 ± 28.81				

*Independent samples *t*-test.

Note: Bonferroni correction applied; adjusted α = 0.0083

## Data Availability

This article adheres to open data practices. The authors will share the raw data upon reasonable request.
